# Graded Motor Imagery (GRAMI Protocol) for Phantom Limb Pain: A Randomised Clinical Trial of Home‐Based Intervention

**DOI:** 10.1002/ejp.70167

**Published:** 2025-11-18

**Authors:** Sandra Rierola‐Fochs, Marc Terradas‐Monllor, Sergi Grau‐Carrión, Mirari Ochandorena‐Acha, Eduard Minobes‐Molina, Jose Antonio Merchán‐Baeza

**Affiliations:** ^1^ Research Group on Methodology, Methods, Models and Outcomes of Health and Social Sciences (M_3_O), Faculty of Health Sciences and Welfare Centre for Health and Social Care Research (CESS), University of Vic‐Central University of Catalonia (UVic‐UCC) Vic Spain; ^2^ Institute for Research and Innovation in Life Sciences and Health in Central Catalonia (IRIS‐CC) Vic Spain; ^3^ Pain Medicine Section, Anaesthesiology Dept, Hospital Clinic de Barcelona Barcelona Spain; ^4^ Digital Care Research Group, Faculty of Science, Technology and Engineering, Centre for Health and Social Care Research University of Vic‐Central University of Catalonia Vic Spain; ^5^ Spanish Society of Geriatrics and Gerontology Madrid Spain

**Keywords:** graded motor imagery, pain, phantom limb pain

## Abstract

**Background:**

Phantom limb pain (PLP) affects 64% of individuals who have undergone amputation. Various theories explain its development, leading to different treatments, including graded motor imagery. This study analyses the effectiveness of a home‐based intervention protocol based on graded motor imagery (GraMI protocol) as a treatment for phantom limb pain.

**Methods:**

A randomised, controlled, home‐based, assessor‐blinded clinical trial was conducted on individuals over 18 years old, with limb amputation, pharmacologically stable and discharged home. Participants followed the GraMI protocol or continued their current treatment for 9 weeks. Assessments were conducted at baseline, postintervention and at 3 months follow‐up, evaluating PLP, quality of life, functionality and depressive symptoms.

**Results:**

The study enrolled 36 participants (mean age of 58.5 years), including 27 individuals with lower limb amputation and nine with upper limb amputation. Vascular issues were the primary cause, and 17 participants experienced preamputation pain. None of the participants in the control group received any PLP treatment during the study. Compliance with treatment among participants in the experimental group during the laterality recognition and explicit motor imagery phases was satisfactory, averaging 91.4%. Significant differences were found between groups in PLP (*p* = 0.02), persisting 12 weeks postintervention (*p* = 0.05). Within‐group analysis revealed clinically significant PLP improvements postintervention (*p* = 0.003), and these improvements remained statistically significant 12 weeks later (*p* = 0.006). There were no statistically significant differences observed in the rest of the variables.

**Conclusion:**

The GraMI protocol shows effectiveness in reducing PLP in individuals who have undergone amputation, with this effect persisting 12 weeks after the intervention.

**Significance Statement:**

Phantom limb pain significantly impacts individuals with amputations, yet effective treatments remain limited. This study is crucial as it evaluates a home‐based graded motor imagery (GraMI) protocol, offering a noninvasive, accessible intervention. The randomised clinical trial demonstrates GraMI's effectiveness in reducing PLP, with lasting effects up to 12 weeks. By addressing PLP, this research contributes to improving patients' quality of life, functionality and psychological well‐being. Its findings support integrating GraMI into rehabilitation programs, providing evidence for a cost‐effective, home‐based therapeutic option.

**Trial Registration:**

ClinicalTrials.gov identifier: NCT05083611

**Abbreviations**
CI: confidence interval
EQ‐5D‐5L: Euro quality of life 5D‐5L questionnaire
FIM: functional independence measure
GMI: graded motor imagery
NNT: number needed to treat
OR: odds ratio
PLP: phantom limb pain
PLS: phantom limb syndrome
SF‐MPQ: short‐form McGill pain questionnaire

## 1 Introduction

Recent years have witnessed a surge in amputations, primarily due to population aging and the rise in traffic accidents (Kaur and Guan 2018). Population aging has led to an increase in poorly managed comorbidities, particularly vascular ones, which are considered the leading causes of amputation (Sugawara et al. 2021; Zheng et al. 2021). Eighty‐five percent of individuals who have undergone an amputation experience phantom limb syndrome (PLS) (Collins et al. 2018). PLS is characterised by the persistent perception of the amputated limb as if it were still present, encompassing both nonpainful and painful sensations (AlMehman et al. 2022; Stankevicius et al. 2021).

Nonpainful sensations encompass all perceptions from the amputated limb except for pain (Beisheim‐Ryan et al. 2021). Painful sensations, known as postamputation pain, include residual pain localised at the stump's proximal part and phantom limb pain (PLP), a neuropathic pain originating in the body area corresponding to the amputated limb (Folch et al. 2022; Sugawara et al. 2021). PLP is characterised by a spectrum of subjective symptoms and affects 64% of individuals after an amputation (Campo‐Prieto and Rodríguez‐Fuentes 2018; Sugawara et al. 2021). Roughly50% of individuals experience PLP within 24 h after amputation (Flahaut et al. 2018; Fuchs et al. 2018), with this percentage rising to 85%–97% within the first month (Pirowska et al. 2014). Despite a potential decrease in frequency and duration over the initial 6 months (Boomgaardt et al. 2022; Eldridge et al. 2021), about 59% of individuals still experience PLP 2 years after amputation (Akbulut et al. 2022), with 15%–20% experiencing it as long‐lasting chronic pain (Eldridge et al. 2021). While the exact pathophysiological mechanisms underlying PLP remain elusive (Boomgaardt et al. 2022; Collins et al. 2018), theories propose a multifactorial origin, with cortical reorganisation gaining prominence (Duarte et al. 2020; Zheng et al. 2021). Maladaptive cortical plasticity following amputation plays a key role in PLP, emphasising the brain's reorganisation as a central factor in the development and persistence of PLP (Makin and Flor 2020).

Graded motor imagery (GMI) is a comprehensive and noninvasive rehabilitation program designed to activate motor cortical networks and aimed at mitigating maladaptive cortical reorganisation postamputation without exacerbating pain (Méndez‐Rebolledo et al. 2017; L. G. Moseley 2004; G. L. Moseley 2006). GMI includes three techniques: laterality recognition or implicit motor imagery, explicit motor imagery and visual feedback‐mirror therapy (Méndez‐Rebolledo et al. 2017; Morales‐Osorio and Mejía 2012; G. L. Moseley 2006; Priganc and Stralka 2011).

Several systematic reviews and meta‐analyses have evaluated the efficacy of GMI and movement representation techniques in chronic pain and PLP, supporting their potential as therapeutic strategies (Bowering et al. 2013; Limakatso et al. 2023; Thieme et al. 2016). However, further studies are needed to validate specific protocols and their applicability in home‐based settings in order to promote adherence to treatment and reduce the need for travel. A systematic review analysed the effectiveness of GMI and its components in managing PLP in amputees (Rierola‐Fochs et al. 2023), laying the foundation for the GraMI protocol—a GMI‐based treatment validated by an expert panel using the Delphi methodology (Rierola‐fochs et al. 2021). This home‐based treatment utilises a mobile application and a mirror box (Rierola‐Fochs et al. 2022), and its feasibility for implementation was studied through a separate feasibility study (Rierola‐Fochs et al. 2024).

This study aims to evaluate the effectiveness of the GraMI protocol in treating PLP in amputees. The secondary objective is to assess the effectiveness of this protocol on improving quality of life, functionality and depressive symptoms.

## 2 Material and Methods

### 2.1 Study Design

A randomised, controlled, home‐based clinical trial with two parallel groups and an assessor blinded was conducted from January to October 2023. This study adhered to the recommendations of the Consolidated Standards of Reporting Trials (CONSORT) guidelines (Eldridge et al. 2016). The study protocol was registered on ClinicalTrials.gov (NCT05083611) and published (Rierola‐Fochs et al. 2022).

### 2.2 Ethical Considerations

This study obtained approval from multiple committees, including the Ethics Committee of the University of Vic‐Central University of Catalonia (UVic‐UCC) with registration number 185/2001, the Ethics Committee on Medicines of the QuirónSalud‐Catalunya Hospital Group with registration number 2022/04‐COT‐ASEPEYO and the Ethics Committee for Clinical Research of the Osona Foundation for Research and Health Education (FORES) with registration number 2022188/PR319. Additionally, the study adhered to the principles outlined in the Helsinki Declaration and complied with Organic Law 3/2018 (December 5), which focuses on Personal Data Protection and Digital Rights Guarantee.

### 2.3 Sample Size

The results from the preceding feasibility study were used as a reference for calculating the required sample size for the current study (Rierola‐Fochs et al. 2024). The primary outcome variable was pain, which was assessed by the visual analogue scale of the Short‐Form McGill Pain Questionnaire (Lovejoy et al. 2014). In that feasibility trial involving 20 participants, it showed an average reduction in pain of 2.85 points with a standard deviation of 1.76, suggesting an effect size of 1.94.

The G*power3 software was utilised, and based on an effect size of 1.94, a power of 0.90 and a significance level (α) of 0.05, it was estimated that six individuals would be required. Considering differences in procedures, treatments, participant profiles and cultural aspects across the five recruitment centres, this estimation was replicated for each centre, aiming to enhance sample representativeness. Since different recruitment centres have variations in procedures, treatments, participant profiles and cultural aspects, this estimation was replicated across the five centres (Andrade 2020). Additionally, factoring in a 20% attrition rate, a total of 36 individuals were estimated (18 per group).

### 2.4 Eligibility Criteria

The established inclusion criteria were individuals aged 18 or older, with either lower or upper limb amputations, with a minimum score of 3/10 on the visual analogue scale of the Short‐Form McGill Pain Questionnaire (SF‐MPQ) (Lovejoy et al. 2014), being pharmacologically stable (no changes in medication type, dosage or frequencies) and who had been discharged from the hospital at the time of recruitment. Those individuals who had visual impairments such as hemianopsia, attention deficits, bilateral amputations or who had previously received treatment with GMI were excluded. Additionally, participants who were in the prosthetic fitting phase during the intervention period were also excluded.

### 2.5 Participants

Participants were recruited from seven different centres in Catalonia, Spain. Among these, there were two second‐level hospitals (Hospital de la Santa Creu de Vic and Hospital de Sant Jaume de Manlleu), one third‐level hospital (Parc Sanitari Pere Virgili de Barcelona), one specialised medical centre in occupational medicine (Asepeyo de Sant Cugat del Vallès), a mutual insurance company (MC Mutual de Barcelona), the Association Sant Jordi (association of amputees in Catalonia) and the ANDRADE association based in Barcelona.

Healthcare personnel at each centre identified individuals who met the inclusion criteria and provided them with information about the current study. Those interested in participating contacted the principal investigator (SRF), who provided a detailed explanation of the study, the procedure to follow and an information sheet. If they agreed to participate, they signed the informed consent. In addition, a withdrawal form was made available in case they wished to discontinue their participation in the study at any time.

### 2.6 Randomisation and Blinding

The randomisation of participants was carried out using a sequence of permuted blocks of four participants with three possible preassigned sequences for both experimental and control groups (Ferreira and Patino 2016). This method ensured an equitable distribution of participants between the experimental and control groups. As participants were recruited, the principal investigator (SRF) allocated them to one of the groups following the established sequence (Ferreira and Patino 2016). This study was assessor blinded, as the assessors responsible for the evaluations did not know to which group each participant belonged. Due to the nature of the intervention and the information provided in the information sheet, participants could not be blinded to the group to which they were assigned.

### 2.7 Interventions

The intervention in this study lasted 9 weeks, followed by a 12‐week postintervention follow‐up phase. The entire process was supervised and conducted exclusively by the principal investigator (SRF), a physiotherapist with a long trajectory of expertise in the field. Following randomisation, all participants, both in the intervention and control groups, received a 30‐min educational session delivered by the principal investigator (SRF). During this session, the physiotherapist explained the physiology of PLP, the principles of brain plasticity and the importance of undergoing rehabilitation.

### 2.8 Control Group Intervention

After the initial educational session, participants assigned to the control group were instructed to maintain their current conventional treatment if they were receiving any. The principal investigator (SRF) contacted them weekly by phone to monitor their progress and record any new activities that might affect PLP.

### 2.9 Experimental Group Intervention

In the experimental group, participants independently performed the GraMI protocol (Rierola‐Fochs et al. 2022) in their homes. This protocol involves three consecutive application techniques.

**FIGURE 1:**
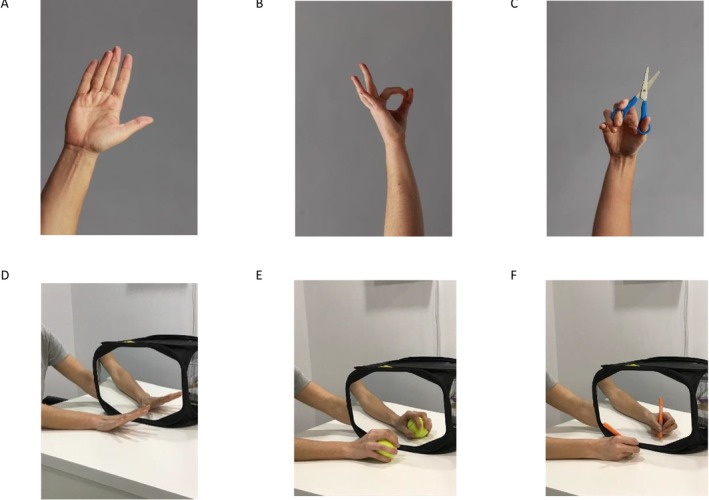
Progression of images during the laterality recognition phase, explicit motor imagery and mirror therapy. (A) Days 1–5 images in neutral positions in different planes; (B) Days 6–10 images in different positions in all planes; (C) Days 11–15 images of activities of daily living or interacting with objects; (D) Days 1–5 analytical movements of the joints involved in the amputation; (E) days 6–10 sensory stimulation; (F) days 11–15 functional activities and interaction with objects.

The first technique is laterality recognition or implicit motor imagery, which was performed during the first 3 weeks of the intervention. It requires participants to quickly distinguish the laterality (right or left) of the two hemi‐bodies through images presented to them. This technique was carried out for 20 min, twice a day, 5 days a week, with a progressive increase in the difficulty of the images over the 3 weeks, as illustrated in Figure 1, following the principles of motor learning.

The second technique is explicit motor imagery, which was performed during the subsequent 3 weeks. In this phase, participants view images in different positions while imagining placing their limb in the same position as the image and returning it to the initial position without performing voluntary contraction, only a mental simulation. Participants were instructed to imagine the movement from a first‐person perspective. Before this phase, the person's imagination capacity was assessed using the *Kinesthetic and Visual Imagery Questionnaire (KVIQ)* (Malouin et al. 2007). During the intervention, individuals with imagination capacity issues received reeducation strategies. This phase also lasted for 20 min, twice a day, 5 days a week, with the difficulty of the images progressively increasing over time, as illustrated in Figure 1. Both techniques were carried out using a mobile application called GraMI, developed by the research group in collaboration with computer engineers. The application recorded several key metrics, including the total compliance of the intervention in percentage, reaction times in seconds, the time required to recognise and confirm an image and the accuracy of correct answers in percentage for each activity during the laterality recognition technique.

The third technique is visual‐mirror feedback therapy, which involves placing a mirror in the sagittal plane between the extremities and positioning the amputated extremity behind the mirror. The person observes the reflection of their healthy extremity in the mirror to teach the brain that pain‐free movement is possible. Additionally, it aimed to preactivate damaged areas to reduce maladaptive changes after amputation (Darnall 2009; Chan et al. 2007). This technique was performed for 20 min, once a day, 5 days a week, for 3 weeks. This technique was also divided into three progressive phases with increasing difficulty, as detailed in Figure 1. Participants needed a mirror of variable size according to their level of amputation and everyday objects to work on sensory stimulation and functional activities. The research group provided all the materials to each participant.

Before each technique, an individual educational session was conducted to explain the objectives, the procedures to follow and to address any questions or concerns. These sessions were delivered face‐to‐face at the participant's home exclusively by the principal investigator (SRF). During the session, the technique and its therapeutic goals were explained in detail, ensuring that participants understood how to perform the exercises correctly. All required materials were provided during these visits. For example, in the session introducing mirror therapy, participants received a mirror box and a kit containing objects for sensory stimulation and interaction with daily‐use items. The duration of these sessions varied depending on the participant's level of understanding and need for clarification, with an average duration of approximately 30 min.

Weekly, the principal investigator (SRF) would follow up with each participant by phone to monitor progress and resolve any issues. In addition, participants had access to the investigator's contact details to ask questions or report any difficulties encountered during the intervention, with the aim of promoting adherence and engagement with the protocol.

### 2.10 Outcomes Measures

Two external researchers (MTM and LAVV) conducted all outcome assessments. These researchers were physiotherapists with expertise in neurorehabilitation and pain management and had prior experience in research methods. They participated in a prior calibration process, which included specific training and standardised instructions to ensure consistent application of the assessment tools. This procedure aimed to guarantee uniform criteria, minimise inter‐rater variability and reduce the risk of measurement bias. Importantly, the assessors were not involved in the delivery or supervision of the intervention. Three assessments were performed: at baseline, at the end of the intervention (9 weeks) and a follow‐up at 12 weeks after the intervention. Each assessment lasted approximately 30–40 min.

### 2.11 Demographic and Health Data

Sociodemographic variables (such as age, gender, marital status, educational level, medical and surgical history, medications, etc.) and characteristics of the amputation (including side, level, cause, duration and progression) were collected in the baseline assessment.

### 2.12 Main Variable

PLP is the primary variable and was assessed through observed and analysed changes using the visual analogue scale of the *Short‐Form McGill Pain Questionnaire* (SF‐McGill Pain Questionnaire) (Lovejoy et al. 2014). SF‐McGill Pain Questionnaire is a self‐administered questionnaire that evaluates pain both qualitatively and quantitatively experienced by the person over the past week. The qualitative section comprises 15 pain descriptors, of which 11 are sensory categories and four are affective. The total score ranges from 0 to 45 points, where higher scores indicate greater pain intensity (Hawker et al. 2011). The quantitative part includes a 100 mm horizontal visual analogue scale for pain, ranging from 0 (no pain) to 100 (worst imaginable pain). A clinically significant reduction in pain was considered when participants decreased their scores by two points per centimetre (Buisman et al. 2022). Overall, this questionnaire has demonstrated high reliability and validity for pain assessment (Ramsay 1994).

### 2.13 Secondary Variables

#### 2.13.1 Quality of Life

The participant's health‐related quality of life was assessed using the Spanish version of the *Euro Quality of Life 5D‐5L questionnaire (EQ‐5D‐5L)* (Herdman et al. 2011). This is a self‐administered questionnaire consisting of two parts. The first part employs a descriptive system to assess health across five dimensions: mobility, self‐care, usual activities, pain/discomfort and anxiety/depression. The total score is converted into a summary index through a descriptive system, ranging from ‘worse than death’ (< 0) to ‘full health’ (1). The second part of the questionnaire includes a visual analogue scale for quality of life, where participants rated their health state on the day of the assessment on a scale from 0 (worst imaginable health) to 100 (best imaginable health). This questionnaire has demonstrated excellent reliability and validity in assessing quality of life (Buchholz et al. 2018).

#### 2.13.2 Functionality

Functionality was assessed using the *Functional Independence Measure (FIM)* scale, which consists of 18 items divided into seven levels. These items encompass personal care, sphincter control, mobility, transfers, walking, communication and social cognition, collectively assessing the participant's motor and cognitive abilities. The scores range from 18 to 136, with higher scores indicating greater independence in performing the associated task for each item (Alves et al. 2019). This scale has shown acceptable reliability and validity for assessing functionality (Ottenbacher et al. 1996).

#### 2.13.3 Depressive Symptoms

Depressive symptoms were assessed using the *Beck Depression Inventory‐II (BDI‐II)* questionnaire (Beck 1961). This questionnaire comprises 21 items designed to detect and evaluate the severity of depression. Scores range from 0 to 63 points, and a score of 20 or higher is indicative of the need for psychological assistance (Beck 1961). This questionnaire has demonstrated excellent reliability and validity in assessing depressive symptoms (Poole et al. 2006).

### 2.14 Data Analysis

Initially, a baseline descriptive analysis of the sociodemographic and health characteristics was conducted, including the median, interquartile ranges for continuous variables and numbers and percentages for categorical variables. Normal distribution of all measures was assessed using the Shapiro–Wilk test (*p* > 0.05), and due to the lack of normal distribution for most variables, the authors used nonparametric statistics. Continuous variables were compared using the Mann–Whitney U test, while categorical variables with two categories were compared using the Chi‐square test, and variables with three or more categories were compared using the linear Chi‐square test.

The main variable in this study was PLP assessed with the visual analogue scale of the SF‐McGill Pain Questionnaire. For the between‐group analysis, the Mann–Whitney U test was used. In the case of intragroup comparisons, a Friedman's ANOVA was computed and the Wilcoxon signed rank test was calculated at the different assessment points. Significance values were adjusted using a Bonferroni correction to control the type I error and enable reliable multiple comparisons. The effect size between groups and within groups was calculated using the following formula:

$$r=\frac{Z}{\surd N}$$

N refers to the total sample size, and Z represents the standardised value by the Mann–Whitney U test for between‐groups comparisons or the Wilcoxon value for within‐groups comparisons (Tomczak and Tomczak 2014). Effect sizes were categorised as none (< 0.2), small (≥ 0.2), medium (≥ 0.5) or large (≥ 0.8) (Keshwani et al. 2021). Additionally, to determine a successful treatment response, a decrease of two points or more on the visual analog scale of the SF‐McGill Pain Questionnaire was established as the criterion. This criterion aligns with literature recommendations, especially in the context of PLP (Buisman et al. 2022). Moreover, a successful response to the GraMI protocol was also assessed by the estimation of the number needed to treat (NNT) at the postintervention and the three‐month follow‐up. Finally, the probability of success between‐groups at the two assessment points was presented through the Odds Ratio (OR).

All statistical analyses were conducted using the SPSS statistical package, version 27.0.

## 3 Results

### 3.1 Participants Characteristics

**FIGURE 2:**
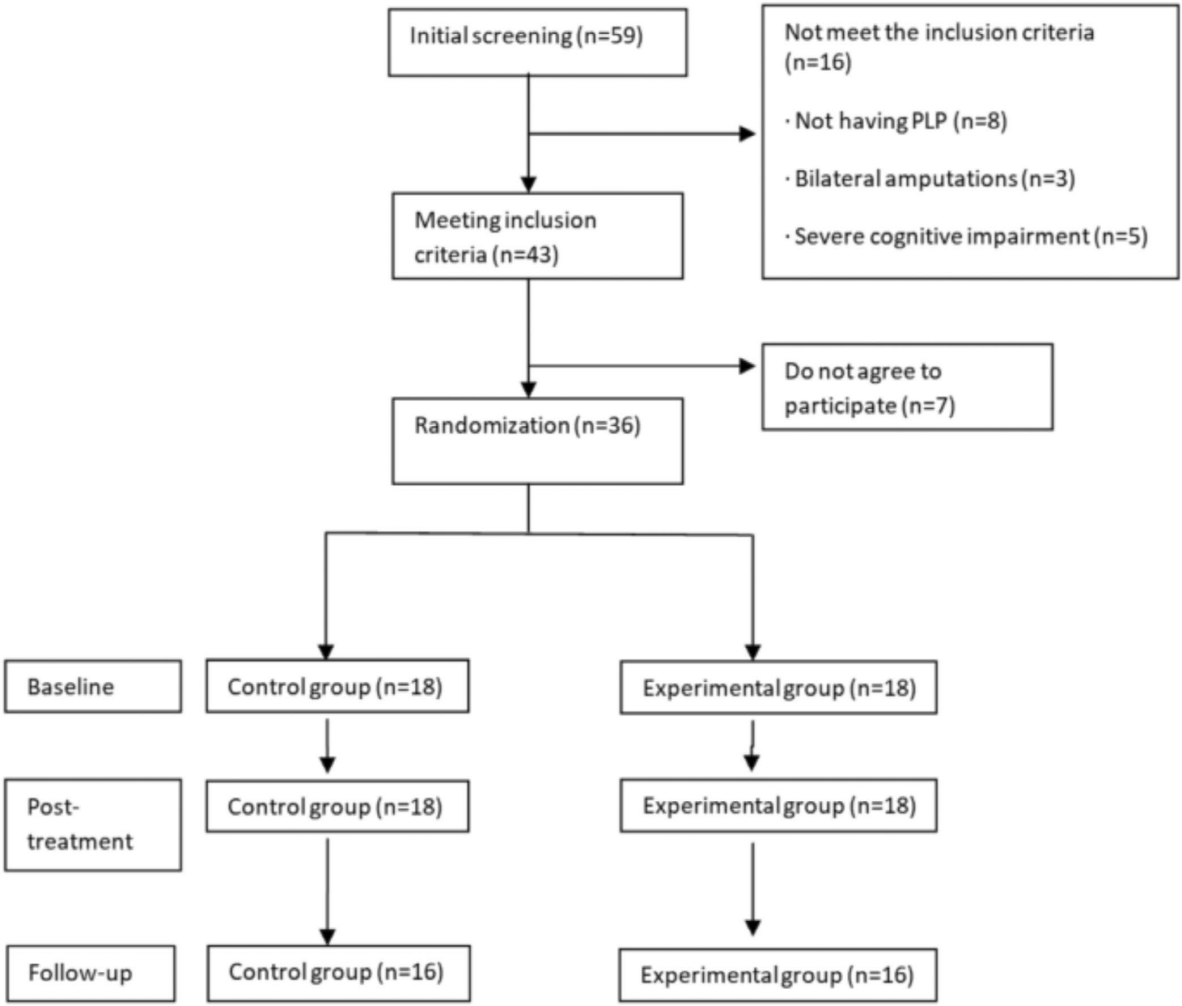
Flowchart. Group assignment and analysis.

Between January and July 2023 (7 months), 59 individuals meeting eligibility criteria were identified. However, only 72.9% (*n* = 43) of these individuals met the established inclusion and exclusion criteria, of which 83.7% (*n* = 36) agreed to participate in the study (Figure 2). Thirty‐six participants commenced the study after randomisation and completed both the initial and the postintervention assessments, which occurred 9 weeks later, without any losses. The follow‐up assessment, conducted 12 weeks postintervention, showed 11.1% (*n* = 4) losses, as shown in Figure 2. Treatment adherence was satisfactory, with an average of 92.4%. All participants (*n* = 36) completed at least 80% of the total 60 proposed activities, although only 10 participants completed 100% of the activities. This ensured the intervention's intensity, frequency and duration as outlined in the protocol for all participants. The sociodemographic and health characteristics variables of all randomised participants can be observed in Table 1. During the initial assessment, the most used descriptors by participants in the control group to describe PLP were discomfort (88.9%), stabbing (88.3%), cramping (72.2%), exhausting (72.2%) and sharp (66.6%). On the other hand, the most used descriptors by participants in the experimental group were discomfort (94.4%), pulsating (88.3%), stabbing (88.3%), sharp (72.2%), cramping (61.1%) and exhausting (61.1%).

**TABLE 1:** Sociodemographic and health characteristics of participants.

Population description	Total (*n* = 36)	Control (*n* = 18)	Experimental (*n* = 18)	*p*
Age [median (IQR)]	58.50 (29–85)	60.17 (29–85)	56.83 (38–78)	0.37^a^
Sex [*n* (%)]				
Male Female	31 (86.1) 5 (13.9)	15 (83.3) 3 (16.7)	16 (88.9) 2 (11.1)	0.63^b^
Civil status [*n* (%)]				
Single Unmarried couple Married Divorced Widow	9 (25) 3 (8.3) 15 (41.7) 6 (16.7) 3 (8.3)	3 (16.7) 0 (0) 9 (50) 3 (16.7) 3 (16.7)	6 (33.3) 3 (16.7) 6 (33.3) 3 (16.7) 0 (0)	0.24^c^
Education level [*n* (%)]				
No studies Elementary Secondary Vocational education Superiors	2 (5.6) 5 (13.9) 12 (33.3) 6 (16.7) 11 (30.6)	2 (11.1) 3 (16.7) 7 (38.9) 2 (11.1) 4 (22.2)	0 (0) 2 (11.1) 5 (27.8) 4 (22.2) 7 (38.9)	0.08^c^
Pathological antecedents [*n* (%)]				
Hypertension Circulation changes Diabetes	14 (38.9) 9 (25) 14 (38.9)	9 (50) 6 (33.3) 9 (50)	5 (27.8) 3 (16.7) 5 (27.8)	
Drugs [*n* (%)]				
Opioids Antidepressants Analgesics Benzodiazepines Anticonvulsants	14 (38.9) 15 (41.7) 14 (38.9) 11 (30.6) 20 (55.6)	8 (44.4) 8 (44.4) 8 (44.4) 7 (38.9) 7 (38.9)	6 (33.3) 7 (38.9) 6 (33.3) 4 (22.2) 13 (72.2)	
Reason of amputation [*n* (%)]				
Vascular Traumatic	21 (58.3) 15 (41.7)	11 (61.1) 7 (38.9)	10 (55.5) 8 (44.5)	0.16^b^
Time since amputation [median (IQR)]				
Months	14 (1–488)	12 (2–57)	16.5 (3–488)	0.24^a^
Site of amputation [*n* (%)]				
Lower limb Upper limb	27 (75) 9 (25)	16 (88.9) 2 (11.1)	11 (61.1) 7 (38.9)	0.50^b^
Level of amputation [*n* (%)]				
Below knee Above knee Below elbow Above elbow	7 (19.4) 19 (52.8) 6 (16.7) 4 (11.1)	4 (22.2) 9 (50) 3 (16.7) 2 (11.1)	3 (16.7) 10 (55.5) 3 (16.7) 2 (11.1)	
Prosthesis [*n* (%)]				
No Yes	15 (41.7) 21 (58.3)	8 (44.4) 10 (55.6)	7 (38.9) 11 (61.1)	0.73^b^
Hours wearing the prosthesis [median (IQR)]	9.38 (1–16)	9.10 (1–12)	9.64 (3–16)	0.86^b^
Preamputationp ain [*n* (%)]				
No Yes	19 (52.8) 17 (47.2)	7 (38.9) 11 (61.1)	12 (66.7) 6 (33.3)	0.09^b^
Onset of PLP [*n* (%)]				
Days after amputation Within the first month of evolution 1–3 months after amputation More than 3 months He doesn't remember	20 (55.6) 7 (19.4) 7 (19.4) 1 (2.8) 1 (2.8)	12 (66.7) 3 (16.7) 1 (5.6) 1 (5.6) 1 (5.6)	8 (44.4) 4 (22.2) 6 (33.3) 0 (0) 0 (0)	0.17^c^

Abbreviations: IQR, Interquartile range; n, number of participants; PLP, phantom limb pain.

^a^ U Mann–Whitney test.

^b^ Pearson's chi‐squared test.

^c^ Linear chi‐squared test.

### 3.2 Intervention Characteristics

None of the participants assigned to the control group underwent any nonpharmacological treatment for PLP during the study. That is, all participants were taking pharmacological treatment for PLP, but aside from that, no additional interventions were provided. Additionally, during the study, participants were pharmacologically stable, meaning there were no changes in their medication dosage. Compliance with the treatment for participants in the experimental group during the laterality recognition and explicit motor imagery phases was satisfactory, with an average of 91.4%. All participants completed a minimum of 80% of the proposed 60 activities, but only two of them completed 100% of the activities. This ensured the intervention's intensity, frequency and duration as described in the protocol for all participants.

### 3.3 Between‐Group Comparison

#### 3.3.1 Main Variable

**FIGURE 3:**
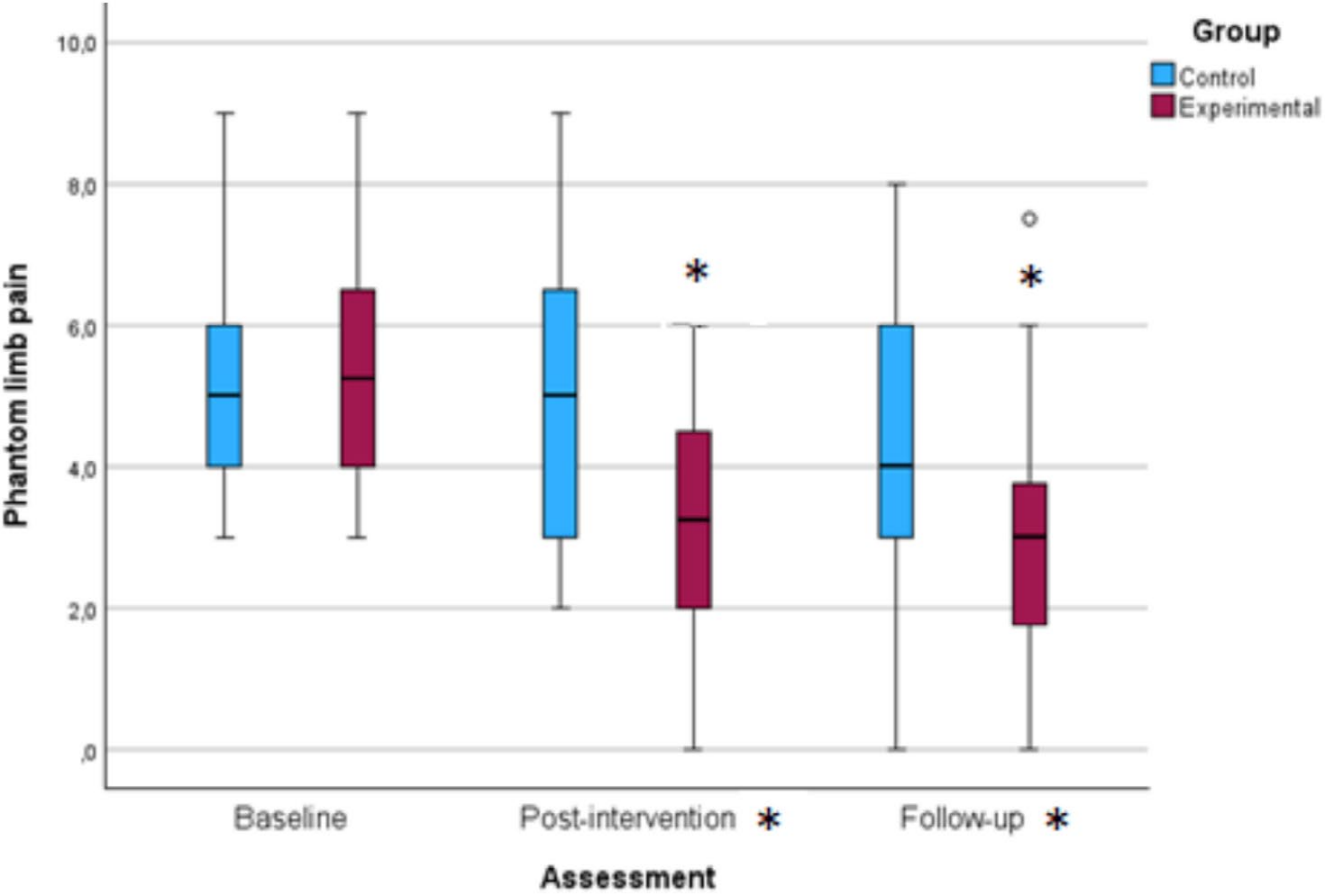
Between‐groups comparison of PLP at each assessment point. *Significant differences between groups and within group.

The primary outcome of the study was PLP, measured using the visual analogue scale of the SF‐McGill Pain Questionnaire. Between‐group comparison showed a statistically significant difference in PLP postintervention (*p* = 0.02), which was maintained at the 12‐week follow‐up assessment (*p* = 0.05) with a ‘small’ size effect. Figure 3 displays the evolution of PLP scores between groups across the three assessment points.

NNT was 2.27 postintervention [95% CI: 2.03–2.51] and 3.03 at the three‐month follow‐up [95% CI: 2.79–3.27]. The probability of success was 7.85 times higher in the experimental group compared to the control group postintervention and four times higher after 12 weeks of follow‐up.

#### 3.3.2 Secondary Variables

No statistically significant differences were found between groups in the secondary variables. A trend towards significance was observed in the quality of life VAS (*p* = 0.054), with a ‘small’ effect size. All other secondary outcomes showed no between‐group differences.

A detailed description of the between‐group analysis results can be observed in Table 2.

**TABLE 2:** Comparative analysis between‐groups.

	Baseline^a^			Posttreatment^b^			Follow up^c^			
	Control (*n* = 18)	Experimental (*n* = 18)	Mann–Whitney U test (*p*)	Control (*n* = 18)	Experimental (*n* = 18)	Mann–Whitney U test (*p*)	Control (*n* = 16)	Experimental (*n* = 16)	Mann–Whitney U test (*p*)	Between‐groups effect size (r)
PLP (SF‐MPQ VAS)	5 (4–6)	5.25 (4–6.63)	0.37	5 (3–6.5)	3.25 (2–4.63)	0.02	4 (3–6.25)	3 (1.62–3.87)	0.05	0.15^a^, 0.08^b^, 0.33^c^
Quality of life index (EQ‐5D‐5L)	0.48 (0.15–0.63)	0.57 (0.37–0.77)	0.08	0.43 (0.20–0.71)	0.65 (0.33–0.77)	0.17	0.53 (0.23–0.73)	0.70 (0.45–0.80)	0.17	0.29^a^, 0.23^b^, 0.23^c^
Quality of life (QOL VAS)	60 (48.75–70)	67.5 (48.75–73.75)	0.44	52.50 (38.75–70)	70 (50–76.25)	0.05	57.50 (50–70)	67.50 (51.25–85)	0.17	0.13^a^, 0.32^b^, 0.23^c^
Functionality (FIM)	112.5 (108–124)	115 (110.75–124.25)	0.33	118 (109.75–120)	118.50 (114.75–123.25)	0.25	118 (112.75–124.75)	115 (113.50–120)	0.55	0.16^a^, 0.19^b^, 0.10^c^
Depressive symptoms (Beck)	13 (8.5–21.25)	16.50 (8.75–28)	0.64	15.50 (8.75–21.25)	14 (8.25–19.25)	0.39	14 (9.25–25.75)	15 (6.50–23)	0.60	0.08^a^, 0.14^b^, 0.09^c^

Abbreviations: EQ‐5D‐5L, Euroqool‐5D‐5LFIM; FIM, Functional Independence Measure; Median (Interquartile Range); *n*, Number of participants; PLP, phantom limb pain; SF‐MPQ, Short Form McGill Pain Questionnaire; VAS, Visual Analogue Scale; *p* value ≤ 0.05 Statistically significant.

^a^ baseline.

^b^ posttreatment.

^c^ follow up.

### 3.4 Intragroup Comparison

In within‐group comparisons, a statistically significant difference in PLP was observed in the experimental group between the initial and postintervention assessments (*p* = 0.003). Moreover, this difference was clinically relevant as there was a reduction of 2 points on the visual analogue scale of the SF‐McGill Pain Questionnaire compared to the initial assessment. This significant difference was also observed between the initial and follow‐up assessments (*p* = 0.006). No significant differences were observed in the rest of the variables.

In the control group, a ‘medium’ effect size was observed between the initial and follow‐up assessments for the quality of life index. The remaining variables exhibited a ‘small’ or ‘none’ effect size in different assessments.

In the experimental group, a ‘large’ effect size was observed for PLP between the initial and post‐intervention assessments, and a ‘medium’ effect size between the initial and follow‐up assessments for PLP, as well as for depressive symptoms between the initial and postintervention assessments. The remaining variables showed a ‘small’ or ‘none’ effect size between different assessments.

A detailed description of the results of the within‐group analysis can be observed in Table 3.

**TABLE 3:** Comparative analysis within‐groups.

		Baseline *p*	Posttreatment	Follow‐up	ANOVA *p*	Within‐groups effect size (r)
PLP (SF‐MPQ VAS)	Control	1	1	1	0.67	0.09^a^, 0.40^b^, 0.22^c^
	Experimental	0.003	1	0.006	0.001	0.86^a^, 0.21^b^, 0.72^c^
Quality of life index (EQ‐5D‐5L)	Control	1	1	0.08	0.29	0.16^a^, 0.21^b^, 0.52^c^
	Experimental	1	1	1	0.87	0.02^a^, 0.12^b^, 0.15^c^
Quality of life (QOL VAS)	Control	0.81	1	1	0.67	0.26^a^, 0.22^b^, 0.03^c^
	Experimental	1	1	0.9	0.28	0.16^a^, 0.16^b^, 0.25^c^
Functionality (FIM)	Control	1	0.93	1	0.75	0.03^a^, 0.23^b^, 0.09^c^
	Experimental	0.96	0.3	1	0.07	0.23^a^, 0.40^b^, 0.20^c^
Depressive symptoms (Beck)	Control	0.42	1	0.24	0.21	0.34^a^, 0.007^b^, 0.41^c^
	Experimental	0.15	1	0.15	0.007	0.67^a^, 0.19^b^, 0.46^c^

Abbreviations: EQ‐5D‐5L, Euroqool‐5D‐5L; FIM, Functional Independence Measure; PLP, phantom limb pain; *p* value ≤ 0.05 statistically significant; SF‐MPQ, Short Form McGill Pain Questionnaire; VAS Visual Analogue Scale.

^a^ baseline.

^b^ posttreatment.

^c^ follow up.

## 4 Discussion

This study aimed to assess the effectiveness of the GraMI protocol in treating PLP in individuals who have undergone amputation. The results suggest that the GraMI protocol is effective in reducing PLP, and this improvement remains statistically significant 12 weeks after the intervention. Furthermore, this reduction in pain is clinically significant.

NNT to achieve a clinically significant reduction in PLP was 2.27 [95% CI: 2.03–2.51], with a success probability of 7.85 times higher in the experimental group compared to the control group after the intervention. The NNT was 3.03 [95% CI: 2.79–3.27] with a success probability four times higher at 12 weeks of follow‐up. These results are in line with previous studies that evaluated the effectiveness of GMI in PLP, showing effectiveness with an NNT of 1.90 [95% CI: 1.11–7.10] and 3 [95% CI: 1.4–10.1] (Limakatso et al. 2020; G. L. Moseley 2006). Additionally, these values are favourable compared to NNTs associated with other treatment options, such as lidocaine 3.80 [95% CI: 1.93–16.61], amitriptyline 5.20 [95% CI: 3.62–9.11] and pregabalin 8 [95% CI: 5.94–32], which are first‐line treatments for PLP (Nurmikko 2008).

The findings of this study also demonstrate a significant reduction in PLP through the implementation of an intensive intervention, involving 5 days per week over 9 weeks, with a gradual progression in task difficulty. These results are supported by previous studies that employed intensive protocols of 7 days per week over 6 weeks, also with an ascending progression in task complexity and reported similar outcomes (Limakatso et al. 2020; G. L. Moseley 2006). While prior research suggests that low‐intensity interventions may not be effective, this study used fewer sessions per week compared to some earlier GMI protocols (Lotze and Moseley 2022; G. L. Moseley 2006). However, the longer duration of each session in this intervention appears to contribute to its effectiveness, indicating that both session length and frequency may play important roles in treatment outcomes.

The GraMI protocol's effectiveness is further highlighted by its intensive nature, which may be a key factor contributing to its efficacy. Sustained and consistent stimulation during intensive protocols likely facilitates cortical reorganisation and supports the underlying neurophysiological processes, thus enhancing the long‐term impact of the intervention. The GraMI protocol is grounded in GMI, comprising three consecutive application techniques: laterality recognition, explicit motor imagery and mirror therapy. These techniques are based on progressive activity incrementation principles (Limakatso et al. 2016), aiming to sequentially activate motor and sensory cortical networks to enhance neural reorganisation without inducing a painful response (Méndez‐Rebolledo et al. 2017). Additionally, it aligns with the theory of cortical reorganisation following amputation (Collins et al. 2018; Duarte et al. 2020).

The effectiveness of the protocol can be attributed to the implementation of a gradual approach, which may facilitate the persistence of obtained benefits and thus consolidate its efficacy in the long term.

The study conducted by Lotze et al. (Lotze and Moseley 2022) suggests that a structured application of the three GMI techniques is more effective in reducing pain than a disordered application. This finding is further supported by Moseley's study, although in that case, the participant profile was patients with Complex Regional Pain Syndrome (L. G. Moseley 2005). Furthermore, mirror therapy has been observed to provide more significant benefits when an individual has a well‐established cerebral schema and a high imaginative capacity (Rierola‐Fochs et al. 2023). Reduction in use or injury, such as the deafferentation caused by amputation, leads to decreased input to the cortex, resulting in a reduction in cortical representation of the amputated body part, accompanied by an expansion of cortical representation of adjacent body parts (Collins et al. 2018). When used in isolation, explicit motor imagery and mirror therapy have the potential to trigger pain responses by inducing neurophysiological activation in brain areas related to the planning and execution of voluntary movement (La Touche et al. 2020, 2012). In this context, the laterality recognition technique plays a pivotal role in preparing subsequent protocol techniques aimed at reorganising cortical body representation (Priganc and Stralka 2011). This specific technique activates the premotor cortex, significantly influencing movement planning and establishing specific connections with the primary motor area responsible for executing movement.

However, implementing the GraMI protocol requires high levels of attention, which can sometimes lead to fatigue, monotony and a lack of motivation (Cuenca‐Martínez et al. 2022). Previous studies have highlighted limitations in participants' adherence to home‐based treatment, affecting the assurance of maintaining the prescribed levels of intensity, frequency and duration of the treatment (Bassett and Smyer 2003; Limakatso et al. 2020). This study demonstrated a favourable adherence, with an average of 91%. This achievement may be associated with digital technologies, as they enable individuals to autonomously engage in interventions, fulfilling a fundamental psychological need for empowerment and active participation in their treatment. Furthermore, digital technologies allow for creating more engaging interventions that enhance adherence and, consequently, treatment compliance (Díaz De León‐Castañeda 2019). In addition to the use of digital tools, continuous telephone contact and face‐to‐face educational sessions conducted before each technique by the research team could have provided personalised support, helping to address any doubts or difficulties and potentially enhancing participant engagement and adherence. Therefore, in this study, the established intensity, frequency and duration in the protocol could be ensured. The focus on treatment adherence may have positively impacted the effectiveness of the GraMI protocol, as previous studies had identified limitations or deficiencies in treatment adherence that could have affected the obtained results (Limakatso et al. 2020).

Nevertheless, no statistically significant differences were found in variables such as quality of life index, analogue visual scale of quality of life, functionality and depressive symptoms. An amputation significantly disrupts an individual's daily life, potentially leading to physical, emotional and social changes. According to a systematic review by Maciver et al. (Maciver et al. 2023), various factors such as gender, age, cause of amputation, level of amputation, ability to use prosthetics, type of prosthetics and phantom limb pain were identified as predictors of quality of life in individuals who have undergone amputation. Additional studies also include factors such as return to work, social support, personality and the time elapsed since the amputation (Calabrese et al. 2023). Therefore, considerable variability in factors influencing the response to amputation can impact an individual's quality of life. However, there are common reactions, including anxiety, reduced quality of life, depressive mood, negative thoughts, pain, concerns about body image and difficulties in social interactions (Eiser et al. 2001). A considerable number of individuals experience dissatisfaction with their body image, which correlates with an increase in depressive symptoms and a decrease in quality of life (Beisheim‐Ryan et al. 2021). Furthermore, emotional disturbances can interfere with the functional recovery of the individual (Calabrese et al. 2023). Consequently, interventions exclusively targeting PLP may not substantially impact the quality of life, functionality and depressive symptoms of these individuals, given the presence of various other influencing factors (Calabrese et al. 2023). For this reason, it is essential to plan a person‐centred rehabilitation or intervention from a biopsychosocial perspective, involving a multidisciplinary team to address the multiple factors that can influence a person's quality of life.

This study marks the first home‐based examination of the effectiveness of a standardised home‐based intervention protocol based on GMI for PLP treatment. The GraMI protocol was designed on the basis of the most recent scientific evidence, motor learning principles, potential solutions addressing limitations observed in previous studies and insights from a panel of national and international experts in neurorehabilitation and pain. The home‐based study was conducted to include individuals with amputations of lower and upper extremities, varying ages, causes and amputation levels to ensure the sample's maximum representativeness. Notably, digital technologies have proven effective as treatment tools, facilitating implementation within communities and reaching all individuals experiencing PLP, thus eliminating the need for travel. Furthermore, this approach might have empowered individuals to play an active role in their own treatment, fostering adherence and motivation. Additionally, leveraging digital technologies enables the monitoring of interventions to analyse compliance and ensure adherence to the parameters established in the protocol. As a conservative and noninvasive treatment, no side effects have been observed in the participants. This study provides healthcare professionals with a new treatment strategy for phantom limb pain.

However, the present study has certain limitations that should be considered. For instance, it did not include a simulated treatment for participants in the control group, impacting the lack of participant blinding and should be considered when interpreting the results. Moreover, this could potentially induce a Hawthorne effect simply by participants being aware that they are involved in a clinical trial, as well as by receiving more attention than the control group (Berthelot et al. 2019). Another limitation involves factors such as treatment refusal or discontinuation by some patients, which could have influenced the observed outcomes. Although this study achieved high adherence rates, these cases cannot be entirely ruled out. Future research should investigate the reasons behind treatment refusal or discontinuation, as well as strategies to optimise adherence and reduce barriers that may limit the GraMI protocol's impact. In addition, although some participants reported reduced use of pain medication and improved accessibility due to the home‐based nature of the intervention, no structured cost‐effectiveness analysis was performed. Future studies should consider incorporating a health economic component to explore potential savings related to medication use, reduced travel to rehabilitation facilities and overall healthcare resource utilisation. Finally, the results are short‐term, and we do not know if they will endure over time. Longer‐term follow‐up studies are needed. A potential future research direction would be to assess the effectiveness of the GraMI protocol in relation to the time since amputation or the duration of phantom limb pain, to determine whether factors such as neuroplasticity or the level of amputation influence the protocol's effectiveness.

## 5 Conclusion

The findings from the current study demonstrate that the GraMI protocol is effective in reducing PLP in individuals who have undergone amputation, and this effectiveness remains significant 12 weeks postintervention. Therefore, it can be considered an effective treatment option for managing PLP.

## Author Contributions

This study was designed by Sandra Rierola‐Fochs, Marc Terradas‐Monllor, Mirari Ochandorena‐Acha, Sergi Grau‐Carrión, Jose Antonio Merchán‐Baeza and Eduard Minobes‐Molina. The experiments were performed by Sandra Rierola‐Fochs, Mirari Ochandorena‐Acha and Marc Terradas‐Monllor. The data were analysed by Sandra Rierola‐Fochs, Jose Antonio Merchán‐Baeza, Marc Terradas‐Monllor, Eduard Minobes‐Molina and Mirari Ochandorena‐Acha, and the results were critically examined by all authors. Sandra Rierola‐Fochs had a primary role in preparing the manuscript, which was edited by Marc Terradas‐Monllor, Mirari Ochandorena‐Acha, Sergi Grau‐Carrión, Jose Antonio Merchán‐Baeza and Eduard Minobes‐Molina. All authors have approved the final version of the manuscript and agree to be accountable for all aspects of the work.

## Conflicts of Interest

The authors declare no conflicts of interest.

## Supporting information


**Table S1:** ejp70167‐sup‐0001‐TableS1.docx.

**Data S1:** ejp70167‐sup‐0002‐Supinfo.pdf.
