# Supplementary material for: Graded Motor Imagery (GRAMI Protocol) for Phantom Limb Pain: A Randomised Clinical Trial of Home‐Based Intervention
Source: Eur J Pain. 2025 Nov 18;30(1):e70167. doi: 10.1002/ejp.70167 (PMC12627984; doi:10.1002/ejp.70167)
Supplement: Supplementary file 1 — Table S1: ejp70167‐sup‐0001‐TableS1.docx. [file EJP-30-0-s001.docx]

|  | Baseline^a^ | | Post-treatment^b^ | | Follow-up^c^ | |
| --- | --- | --- | --- | --- | --- | --- |
|  | Control  (n=18) | Experimental (n=18) | Control  (n=18) | Experimental  (n=18) | Control (n=16) | Experimental (n=16) |
| PLP (SF-MPQ VAS) | 5.29 (4.26-5.74) | 5.29 (4.28-6.22) | 4.83 (3.71-6.29) | 3.29 (2.28-4.22) | 4.42 (2.73-5.27) | 2.83 (2.12-3.88) |
| Quality of life index (EQ-5D-5L) | 0.42 (0.30-0.66) | 0.57 (0.42-0.72) | 0.45 (0.24-0.62) | 0.58 (0.49-0.81) | 0.50 (0.33-0.73) | 0.65 (0.56-0.84) |
| Quality of life (QOL VAS) | 59.58 (52.13-67.87) | 63.33 (58.26-76.74) | 53.75 (40.93-64.07) | 65.42 (60.29-79.71) | 59.17 (49.65-65.35) | 67.92 (54.25-80.75) |
| Functionality (FIM) | 114.83 (106.58-118.42) | 116.67 (110.01-119.99) | 115.92 (114.20-121.80) | 118.83 (115.36-121.64) | 118.50 (113.29-122.71) | 116.2 (112.45-117.55) |
| Depressive symptoms (Beck) | 14.3 (8.27-17.73) | 17.8 (9.38-23.62) | 15.2 (10.87-20.13) | 13.8 (9.93-18.07) | 16.3 (7.52-20.48) | 14.8 (8.52-21.48) |

Table S1.

Mean (confidence interval 95%); n Number of participants; PLP phantom limb pain; SF-MPQ Short Form McGill Pain Questionnaire; VAS Visual Analogue Scale; EQ-5D-5L Euroqool-5D-5L; FIM Functional Independence Measure
